# A Secret of Success

**Published:** 1888-02

**Authors:** 


### A Secret of Success.

In an interesting paper read before the New Hampshire Medical Society, this year, Dr. W. S. Leonard points out a number of reasons why the laity come to trust in the virtues of non-professional prescriptions, and neglect to seek the advice of their physicians. One of these reasons, to which attention may be profitably called, is the habit many medical men have of treating slight ailments slightingly. This habit is often strongest in the ablest practitioners, who conscientiously believe that it is only honest to show a patient with a trivial ailment that it is trivial. But we believe that it is rarely a good service to do this. As the physician's object is to cure, he must study what course is best adapted to this end; and if this be to keep to himself the belief that his patient is exaggerating the importance of his trouble, it is neither kind, nor especially virtuous, to disclose his opinion. This is a very different thing from practicing upon, and increasing, a patient's fears—the refuge of none but dishonorable men. It is simply showing proper consideration for the state of mind in which the patient is, and securing his harmonious cooperation in the work of cure.

An extreme of candor is harmful in many cases, as it rarely leads the patient to distrust his own opinion, and rarely raises his appreciation of the doctor's. More than this, the habit of treating hastily or superficially ailments which, to the physician, seem of little consequence, often leads patients to think that the physician despises cases which do not call for the greatest skill, or present features of rare interest. Dr. Leonard quotes a man with a lame knee, who came to an old physician whom he had tried in vain to interest in his case, and said: "Doctor, a man must come to you with his shroud in his hand, before you will do any thing for him."

When the community thinks thus of a physician, his usefulness, as well as his prosperity, is in imminent danger. Both these are proper objects of solicitude for the right-minded physician; and for the sake of both he should not despise the lesser ills of his patients, remembering—besides the fact that what seems trifling may really be serious—that no ill seems small to the one who bears it, and that an honest and kindly sympathy for small ailments and little anxieties is the mark of a large heart and a wise head.

